# Ephaptic conduction in tonic–clonic seizures

**DOI:** 10.3389/fneur.2024.1477174

**Published:** 2024-11-29

**Authors:** Avinoam Rabinovitch, Revital Rabinovitch, Ella Smolik, Yaacov Biton, Doron Braunstein

**Affiliations:** ^1^Department of Physics, Ben-Gurion University, Beer-Sheva, Israel; ^2^Makif YudAlef, Rishon Lezion, Israel; ^3^Department of Physics, Sami Shamoon College of Engineering, Beer-Sheva, Israel

**Keywords:** ephaptic, tonic–clonic, seizures, EEG, cellular automaton (CA)

## Abstract

**Objectives:**

Electroencephalograms (EEGs) or multi-unit activities (MUAs) of tonic–clonic seizures typically exhibit a distinct structure. After a preliminary phase (DC shift, spikes), the tonic phase is characterized by synchronized activity of numerous neurons, followed by the clonic phase, marked by a periodic sequence of spikes. However, the mechanisms underlying the transition from tonic to clonic phases remain poorly understood.

**Methods:**

We employ a simple two-dimensional cellular automaton model to simulate seizure activity, specifically focusing on replicating the tonic–clonic transition. This model effectively illustrates the physical processes during the ictal phase and, more importantly, differentiates the roles of neurons’ activity, identifying their origin as either synaptic or ephaptic.

**Results:**

Our model reveals an intriguing interaction between the synaptic and ephaptic modes of action potential wave conduction. By replicating the EEG and multi-unit activity (MUA) structure of a tonic–clonic seizure and comparing it with real MUA data, we validate the model’s underlying assumption: the transition from tonic to clonic phases is driven by a shift in dominance from synaptic to ephaptic conduction. During synaptic-mode control, neural conduction occurs through synaptic transmission involving chemical substances, while in the ephaptic mode, information transfer occurs through direct Ohmic conduction.

**Significance:**

Gaining a deeper understanding of the neuronal electrical conduction transitions during tonic–clonic seizures is crucial for improving the treatment of this debilitating condition.

## Introduction

A tonic–clonic, historically referred to as “grand mal,” seizure, is one of the most common seizure types appearing in epilepsy. The key differences between these phases are as follows: The tonic stage involves the sudden contraction of muscles, leading to stiffness or muscle rigidity. The muscles contract uncontrollably, resulting in sustained rigidity, typically affecting the arms, legs, or body. Symptoms include a stiffened body, and the individual may fall if standing. Breathing can be compromised, potentially causing cyanosis (a bluish skin discoloration) due to oxygen deprivation. Consciousness is often impaired, and the average duration of the tonic phase is ~20 s. The clonic part, on the other hand, is characterized by rhythmic, repetitive muscle movements. Unlike the tonic stage, which causes sustained muscle contraction, the clonic one involves alternating muscle relaxation and contraction cycles, including rhythmic, repetitive jerking or twitching movements of the arms, legs, or face, typically in a consistent pattern. The average tonic–clonic seizure duration is ~1 min.

Over the years, several hypotheses have been proposed to explain the transition from the tonic to the clonic phase in seizures. One widely accepted theory, based on Lothman’s work (1), is presented in Kandel’s Principles of Neural Science (2): “As the GABA-mediated inhibition gradually returns, the neurons in the seizure focus enter a period of oscillation corresponding to the clonic phase.” However, this hypothesis lacks a detailed mechanism for how such inhibition returns. Furthermore, as demonstrated in our model, inhibition suppresses rather than facilitates wave propagation, making this explanation tenuous. Another proposed mechanism is the slow afterhyperpolarization (sAHP) model (3), based on the observation that, following bursts of action potentials, neurons may enter a period of sAHP, leading to spike-frequency adaptation (4). We propose that this sAHP behavior could be a manifestation of the ephaptic domination phase (see below). Although some mathematical models (5) attribute the transition to small parameter changes, the underlying mechanism remains inadequately understood. Thus, a more comprehensive understanding of this transition is needed, and we aim to provide a novel explanation.

In tonic–clonic seizures, the EEG or MUA during the tonic phase shows highly synchronized activity [e.g., (6)], albeit with a chaotic appearance potentially modeled by a modified Kuramoto system (7), whereas measurements during the clonic phase display periodic oscillations. These clonic oscillations are considered ectopic (appearing only during seizures) and are distinct from the brain’s intrinsic oscillations, such as α and β waves, which are generated by built-in feedback systems like the thalamocortical loop (8, 9). Unlike intrinsic oscillations, ectopic waves lack neuronal feedback mechanisms and could spread unhindered in a purely excitatory medium, leading to the observed periodic clonic oscillations. However, in the normal cortex, inhibitory (GABAergic) neurons prevent such spread through synaptic action potential conduction. Furthermore, the propagation speeds of clonic epileptiform waves do not match typical synaptic conduction velocities.

We suggest that these ectopic clonic oscillations are unlikely to propagate through regular synaptic neural conduction. Instead, we propose that the brain’s conduction mode during the clonic phase is non-synaptic, or possibly a hybrid of synaptic and non-synaptic modes. There are three recognized non-synaptic brain conduction modes: ephaptic conduction, ionic diffusion, and axonal conduction [(10, 11) and references therein]. Since the wave velocities associated with ionic diffusion and axonal conduction do not match the speeds of epileptiform activity observed in animal models, it is plausible that ephaptic conduction governs the clonic phase. In this mode, neurons are activated not by synaptic transmission but by the electric fields generated by nearby neurons. Under normal conditions, these electric fields are too weak to trigger ephaptic conduction. However, during the tonic phase of a seizure, the intense neuronal activity generates significantly stronger electric fields, which may transform (10) the brain’s normal excitatory-inhibitory synaptic operation into one dominated by ephaptic conduction. This ephaptic mode could propagate waves at velocities matching those observed in clonic seizures, with or without assistance from synaptic conduction.

In this study, we develop a two-dimensional cellular automaton (CA) model to explore the fundamental biophysical processes underlying the transition from the tonic to the clonic phase in seizures. Our model examines the propagation of action potential waves under both synaptic and ephaptic conduction modes and demonstrates the shift from synaptic to ephaptic domination during a tonic–clonic seizure.

## The model

Ephaptic behavior in the brain [(12) and related works] has been modeled through highly complex mathematical frameworks. However, to address this phenomenon more intuitively, we propose using a cellular automaton (CA) model. This approach allows us to differentiate between neurons activated via synaptic conduction and those influenced by the ephaptic mechanism, enabling a clearer understanding of the fundamental physical processes during the clonic phase of a seizure.

Our model comprises an N × N grid of cells, representing neurons within a neural network (NS). Each cell, denoted as C, can exist in one of three forms: active (1), indicating that the neuron is firing an action potential; ready or waiting (0), indicating that the neuron is primed for activation; and refractory (2, 3, etc.), indicating that the neuron cannot be activated. The interactions between these cells are governed by synaptic and ephaptic mechanisms, as described below.

### Synaptic mode

In normal brain function, the transmission of electrical signals occurs primarily through synapses, where action potentials propagate from one neuron to another. Within this context, neurons can be classified into two primary types based on their function: excitatory and inhibitory. Excitatory neurons promote the activation of neighboring neurons, while inhibitory neurons reduce or suppress this activation. The inhibitory neurons are distributed randomly throughout the neural matrix, with their proportion denoted by the inhibitory neuron percentage (INP), which reflects their prevalence in the overall neural network. In our model, we define a generation length, referred to as a time unit (TU), to be approximately 1 millisecond (ms), which represents the average duration of a normal action potential. We assume that an active neuron, or “form 1,” remains in this state for one TU. The ready state, “form 0,” persists indefinitely unless the neuron is activated, and the duration of the refractory form (RD) is controlled by the model, with the RD varying over a range of TUs based on our parameters.

The transition of neurons from one state to another is governed by specific rules. The system progresses from generation n to generation n + 1 as follows:

1. Active Cells: Active Neurons (labeled as “1”) in generation n will always transition to the refractory form in generation n + 1, where their label changes to “2.”
2. Refractory Cells: Neurons already in the refractory form in generation n will continue in this state, as long as their refractory period has not expired. The label of a cell will increment by 1 with each successive generation, moving from “2” to “3,” from “3” to “4,” and so on. Once the refractory period ends, the neuron returns to the ready form, labeled “0.”
3. Ready Cells: Neurons in the ready form (labeled “0”) in generation n can become active (labeled “1”) in generation n + 1 based on a probability, *p*. This activation occurs if the sum of the excitatory inputs from the surrounding active neurons surpasses a specific threshold, represented mathematically as:

(1) $$\underset{i}{\sum }f_{i}\geq 1$$

where the index *i* iterates over all active neighboring cells, and *fi* represents their influence. Excitatory neighbors contribute *fi* = +1, while inhibitory neighbors contribute *fi* = −1.

Given that our model is designed, specifically, to investigate neuronal dynamics during tonic–clonic seizures, we simulate the shift from normal brain activity to seizure-like activity by adjusting the system’s excitability parameter, *p*. In normal brain function, the probability of a neuron becoming active, *p*, remains less than 1. However, during epileptic conditions, excitability increases significantly, and we model this by setting *p* = 1, reflecting the heightened likelihood of neuronal firing at the onset of a seizure. This adjustment allows us to examine how the model behaves under both typical and epileptic conditions, providing insights into the mechanisms that underlie seizure initiation and propagation.

### Ephaptic mode

In the ephaptic conduction mode, neurons interact not through traditional synaptic connections but via the electric fields generated by nearby active neurons. This form of interaction is governed by the electric potentials created by the currents flowing through adjacent neurons, influencing the membrane potential of a nearby ready neuron. The effect of ephaptic conduction can be modeled using the quasi-static approximation of Maxwell’s equations (13). Under this approximation, the potential *ϕ* at a distance *r* from an active neuron is described by the equation:

ϕ=aI/4πr

where *a* is a constant that depends on the neuronal resistance and tissue properties (14), and *I* is the current in the active neuron.

In this framework, a neuron in the ready form (denoted as a “0” cell) becomes active if the cumulative effect of the electric potentials from surrounding active neurons (denoted as “1” cells) exceeds a threshold *b*. The contribution from each active neuron at a distance *r_i_* is given by:

$$A_{i}=1/r_{i}$$

where *r_i_* is the normalized distance between the *i*-th active neuron and the ready neuron. For example, the distance to the nearest neighbors is $r_{\mathrm{nearest}\;\mathrm{neigbors}}=1$ , while the distance to the next-nearest neighbors is $r_{\mathrm{next}\;\mathrm{neigbors}}=\sqrt{2}$ , and so on.

The activation condition for a ready neuron is met when the sum of the contributions from all surrounding active neurons satisfies the inequality:


(2) $$\underset{i}{\sum }A_{i}\geq b$$

where *i* refers to all active neurons within a predefined radius *R* from the ready neuron. The threshold *b* is a normalization constant that incorporates several factors, including the amplitude of the current *I*, the actual intercellular distance, and the physiological activation threshold. For simplicity, we assume *b* is identical for all neurons in the system.

In our model, we tested different values of *b* and determined that a threshold value of 2.0 was appropriate for triggering activation only when at least three active neurons are within the “vicinity” of the ready neuron. Instead of using a circular region with radius R, we approximate the neighborhood by considering a 3×3 grid of cells surrounding the ready neuron. This grid-based approach simplifies the computation of interactions while maintaining the essential characteristics of ephaptic conduction in a localized neuronal environment.

The threshold condition in Equation 2 thus provides a mechanism for modeling ephaptic activation, capturing the influence of nearby active neurons through electric field effects rather than direct synaptic input.

### Combined mode

When the brain operates under both synaptic and ephaptic modes of conduction, a neuron labeled “0” in generation n will become active (labeled “1”) in generation n + 1 if either the synaptic condition (1), the ephaptic condition 2, or both are satisfied. Given that ephaptic influence propagates electromagnetically at a very fast speed, condition 2 is considered first. However, considering that the velocity of ephaptic waves, $v_{e}$ , ranges between 0.1 m/s and 0.3 m/s (15, 16), and that the velocity of synaptic transmission $v_{s}$ , is typically higher, ranging from 0.5 m/s to 100 m/s, we introduce a delay *d* in our model to account for the slower ephaptic conduction relative to synaptic transmission (17).

This delay *d* represents the number of generations between the detection of ephaptic activation and its actual occurrence. For instance, if we assume that $v_{s}=2v_{e}$ , then the delay *d* = 1, and if $v_{s}=3v_{e}$ , then *d* = 2, and so on. The delay is implemented by temporarily labeling a neuron that satisfies the ephaptic activation condition with a distinct number (e.g., 6, 7, etc.), rather than immediately transitioning it to the active form (labeled “1”). The concrete transition to the active form occurs only after *d* generations.

For example, if *d* = 1, the ephaptic activation sequence would be: 0 → 6 → 1 → 2 → 3, whereas for *d* = 2, the sequence would be: 0 → 6 → 7 → 1 → 2 → 3. This delay reflects the slower propagation of ephaptic waves relative to synaptic transmission. Importantly, the speed at which the signal instructing the ready neuron to become active travels is instantaneous (at the speed of light), while the ephaptic wave responsible for triggering the neuronal activation is much slower (see discussion).

In our model, the initiation of ephaptic activity in a neuron is marked by a transition of its label from “0” to “6.” Therefore, the number of cells labeled “6” at any given time, provides an estimate of the neurons that will be activated via ephaptic conduction in future generations. By tracking both the number of active cells (labeled “1”) and the number of future ephaptic cells (labeled “6”) throughout the matrix, we can monitor the dynamics of neuronal activation as a function of time (measured in generations). This approach allows us to examine the interplay between synaptic and ephaptic modes of neuronal communication and their impact on brain function over time.

### Electrodes for multi-unit activity

To simulate multi-unit activity (MUA), we define a 6 × 6 grid of cells centered at specific points in the matrix. The number of active cells (labeled “1”) and cells pending ephaptic activation (labeled “6”) within this grid are tracked over time, providing a measure of the progression of ephaptic activity during a seizure.

This model allows for the examination of action potential wave propagation and the interaction between synaptic and ephaptic mechanisms during the tonic–clonic transition, offering insight into the underlying biophysical processes of seizure dynamics.

## Results

### Validation of model dynamics

#### Synaptic conduction excitability

To accurately simulate brain activity during an epileptic seizure, our model must capture the excitability enhancement characteristic observed at seizure onset. In the synaptic conduction mode of our model, neuronal excitability is modulated by the probability *p*, which governs the likelihood of a neuron transitioning from a resting to an active state, provided the condition outlined in Equation 1 is met.

To assess the influence of the excitability parameter *p* on the system, we conducted simulations using 20 independent matrices of dimensions 30 × 30, with 20% of the neurons designated as inhibitory cells distributed randomly throughout each matrix. The simulations began with 20 active neurons operating simultaneously. The overall excitability of the system was quantified by measuring the average number of neurons remaining active in the matrix after 500 TUs.

**Figure 1:**
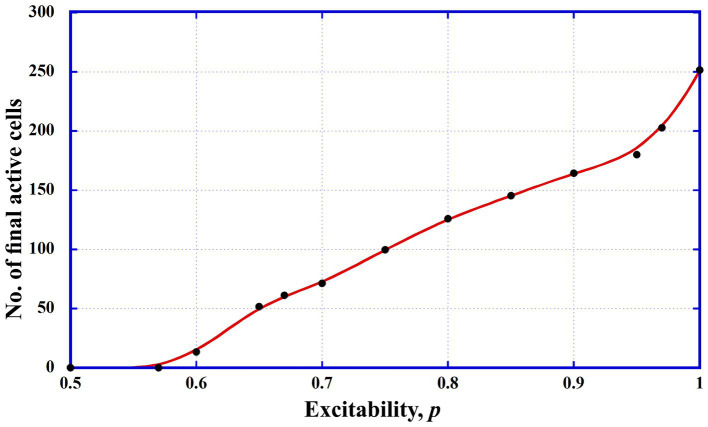
Model excitability curve. In our model, excitability is related to the percent probability *p* of inducing a cell to operate if the conditions of the adjoining cells are ripe (Equation 1). It is measured by the final number of operating cells in the matrix, starting with 20 operating ones. Figure indicates the brain conditions: no function (*p* < ~0.6); regular functioning (~0.65 < *p* < ~0.8), and epileptic functioning (*p* > ~0.9).

The results, shown in Figure 1, depict the average number of active neurons after the simulations across different values of *p*. A critical observation is that when *p* is below approximately 0.6, the system fails to maintain activity, with no neurons remaining active in the matrix. This threshold suggests, that, for excitability levels below a certain point, the system cannot sustain neuronal activation.

The results show two distinct, sharp increases in the curve: one around *p* = 0.6, where the final number of active neurons rises from 0 to approximately 20, and a second, more pronounced rise above *p* = 0.95. Based on this, we propose that in our model, excitability values between *p* = 0.65 and *p* = 0.8 correspond to normal brain function, while values exceeding *p* = 0.9 to *p* = 0.95 reflect conditions that could trigger epileptic seizures.

For the subsequent sections of this study, we focus exclusively on the epileptic seizure state and, therefore, set the excitability parameter to *p* = 1.

#### Excitability under combined synaptic and ephaptic conduction

To evaluate the behavior of our system under both ephaptic and synaptic conduction modes, we conducted simulations using four different excitability values for synaptic conduction (denoted by *p*): 0.6, 0.7, 0.85, and 1. Synaptic activity was governed by condition (1), while ephaptic conduction followed the rules outlined in condition 2. In each case, we ran simulations on 10 matrices of size 30 × 30, with 20% of the neurons being inhibitory, *d* = 12 TUs and 50 cells randomly initialized in the active state. The system’s dynamics were observed throughout 2000 TUs to allow for stabilization into a steady state.

The results (Table 1) indicate a significant difference in neuronal activity depending on the excitability parameter *p*. For the case of *p* = 1, representing an epileptic state, the system stabilized with approximately 200 operational neurons in the steady state, signifying a high level of neuronal activity consistent with epileptic seizure-like behavior. Conversely, for *p* = 0.7, indicative of normal brain function, the number of operational neurons in the steady state was considerably lower, reflecting a more typical and regulated level of neuronal firing.

**Table 1:** Final neuronal activity under combined synaptic and ephaptic conduction as a function of excitability (*p*).

Excitability, *p*	Final activity, FA
0.6	0
0.7	51
0.85	110
1	204

FA represents the average number of active cells during the last 10 TUs out of 2000 TUs, across 10 simulations using 30 × 30 matrices with 20% inhibitory neurons and an initial activation of 50 randomly selected cells.

These findings underscore the system’s sensitivity to excitability changes and demonstrate that, under combined ephaptic and synaptic conduction, the parameter *p* plays a critical role in determining whether the brain operates under normal or epileptic-like conditions.

#### Non-intrinsic (ectopic) waves in synaptic conduction

**Figure 2:**
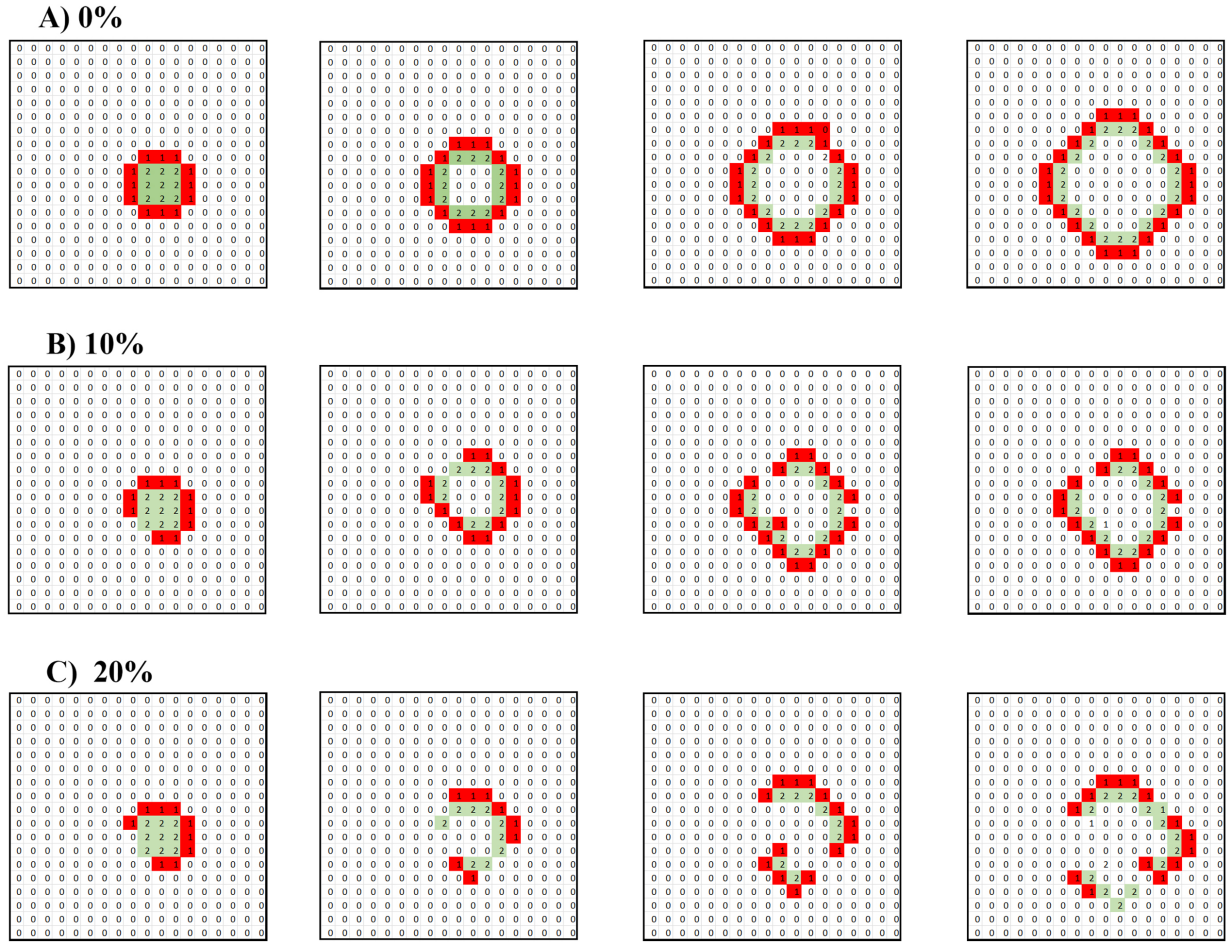
Disruption of a target wave under synaptic conduction by 10 and 20% inhibitory neurons. A 2 × 2 unit of operating cells is introduced into a matrix under pure synaptic conduction. **(A)** Only excitatory cells. **(B)** Randomly distributed 10% of inhibitory cells. **(C)** Randomly distributed 20% of inhibitory cells. The drawings follow the time propagation of the ensuing waves.

We investigated the emergence and behavior of non-intrinsic, or ectopic, waves in systems governed purely by synaptic conduction. The primary focus was how these waves propagate in purely excitatory environments and in systems containing inhibitory cells. Our results (Figure 2) reveal two key phenomena:

(1) Propagation in Purely Excitatory Media: In a system devoid of inhibitory cells, where all neurons were excitatory, a single full target wave was initiated by briefly activating a grid of neurons for one TU. This initial perturbation caused a concentric (target) wave to propagate outward from the activated region, expanding across the matrix. As expected, the wavefront grew steadily in size, maintaining its form as it traveled through the network, until it eventually reached the boundaries of the matrix, where it dissipated due to the lack of further available space for propagation. This behavior is characteristic of excitable media in which there is no opposition to the wave’s spread.
(2) Disruption in Mixed Systems with Inhibitory Cells: When 10 and 20% of the cells in the matrix were inhibitory, the propagation dynamics of the wave were notably altered. Instead of maintaining a coherent structure, the initially formed wave was disrupted by the presence of inhibitory cells distributed randomly throughout the system. As the wave encountered these inhibitory cells, its progression was interrupted, causing fragmentation, and resulting in a breakdown of the organized wave into a scattered pattern of operational cells, where activity no longer propagated smoothly or predictably. Instead, isolated clusters of active cells emerged, reflecting a more disordered and less sustained form of neuronal activity.

These findings illustrate the critical role that inhibitory cells play in modulating the propagation of neuronal waves. In purely excitatory environments, waves can propagate freely, whereas the introduction of inhibitory cells creates barriers that fragment and suppress wave activity. This dynamic highlights the importance of balance between excitatory and inhibitory forces in maintaining regulated neuronal behavior and preventing the unchecked spread of excitatory waves, which could lead to pathological conditions such as seizures.

#### Non-intrinsic waves in ephaptic conduction

In our investigation of non-intrinsic waves within a matrix characterized by pure ephaptic conductivity, we initialized the system with a grid of cells activated for 1 TU. The focus of this experimental setup was to explore the behavior of neuronal activity under conditions solely influenced by ephaptic interactions, without the modulation of synaptic transmission.

Results of these simulations, although not presented here in detail, revealed an intriguing outcome: rather than the formation of propagating waves as observed in systems exhibiting synaptic conduction, the ephaptic configuration resulted in the emergence of a completely periodic pattern. This periodicity was marked by a regular sequence of activations and deactivations that repeated every two delaying periods, signifying a form of stable oscillatory behavior.

The absence of wave propagation in the ephaptic mode can be attributed to the fundamental nature of ephaptic interactions, which rely on the electric fields generated by neighboring active neurons rather than traditional synaptic signaling mechanisms. As such, the neuronal communication in this mode is inherently localized, leading to synchronized activity across cells without the requisite transmission of a wavefront.

This observation has significant implications for our understanding of neuronal dynamics in ephaptic conduction, suggesting that, under certain conditions, neuronal networks may exhibit behavior characterized by periodic oscillations rather than the traveling waves that are commonly associated with excitatory synaptic activity. Future studies will aim to further elucidate the parameters influencing this periodic behavior, including the role of cell density, intercellular distances, and the electrical properties of the cells involved, to better understand the mechanisms governing ephaptic communication and its potential physiological relevance.

### Tonic–clonic seizure

**Figure 3:**
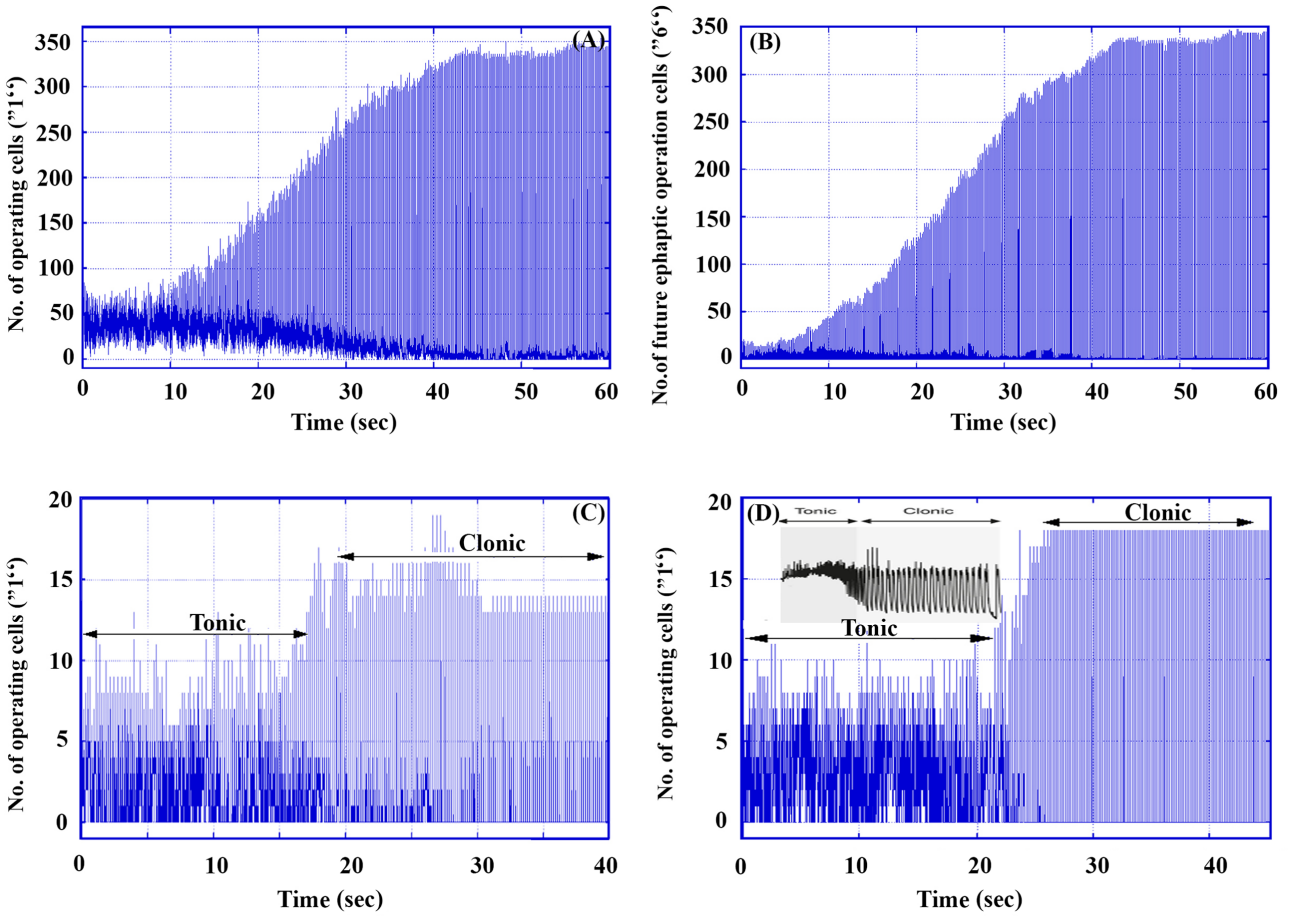
An example of a tonic–clonic seizure in a 30 × 30 matrix under high excitability (*p* = 1). **(A)** The number of total operating cells (labeled “1”) in the whole matrix as a function of time. **(B)** The number of future ephaptic mode operating neurons (labeled “6”) in the whole matrix as a function of time. **(B)** Total operating cells in an electrode of 6 × 6 cells located at (7, 7). **(D)** Total operating cells in an electrode of 6 × 6 cells located at (20, 20). **(D)** Inset. An example of a single neuron response in a real tonic–clonic seizure [Figure 1 in Raimondo JV, Burman RJ, Katz AA, and Akerman CJ Ion dynamics during seizures. Front. Cell. Neurosci. 9, 419, (2015). doi: 10.3389/fncel.2015.0041].

Under high excitatory conditions, where *p* = 1, Figure 3 presents data from a single simulation run over 60,000 TUs (60 s) for a 30 × 30 matrix containing 80% excitatory and 20% inhibitory neurons. Starting with 50 randomly activated cells (labeled “1”), the graphs illustrate key aspects of neuronal activity.

Figure 3A shows the total number of active neurons (operating cells) as a function of time, while Figure 3B depicts the number of cells that will engage in ephaptic mode activity in future generations, providing insight into the evolution of ephaptic interactions in the system. The simulation uses a delay parameter *d* = 160, accounting for the time lag between ephaptic influence and the actual activation of cells. Note that this change leads also to increased cell activity.

Additionally, Figures 3C,D represent the simulated operations of two Multi-Unit Activity electrodes placed at different positions within the matrix. These traces capture local neuronal activity, illustrating the dynamics of neuronal firing. For comparison, the inset in Figure 3D shows an actual recorded trace of a single neuron, highlighting similarities in the signal patterns between the model and real-world brain activity.

This comprehensive simulation provides a clear depiction of both synaptic and ephaptic contributions to neural operation under epileptic conditions.

Numerical calculations in the model effectively capture the key characteristics of real EEG patterns during the transition phase of a tonic–clonic seizure. An actual tonic–clonic seizure, as shown in the inset of Figures 3C,D typically lasts around 1 min. In contrast, the model focuses primarily on the transition phase rather than the entire seizure process. The model highlights two distinct stages that resemble those observed in real seizures (as seen, for example, in Figure 3A): an initial chaotic stage with low-amplitude fluctuations corresponding to the tonic phase, lasting ~20 s (20,000 TUs), followed by a more ordered stage of low frequency and higher amplitude, representing the clonic phase. In the chaotic tonic phase, the number of operating cells hovers around 60, while during the clonic phase, *periodic oscillations* occur between 0 and 350 operating cells. Notably, this transition from tonic to clonic behavior occurs naturally within the model without requiring external parameter adjustments.

The model does not extend to the final phase of the seizure, which typically involves a gradual decrease in frequency and subsequent termination. However, it does capture the main elements of the seizure transition.

Figures 3A–D offer detailed insights into the dynamics of the transition between synaptic and ephaptic modes during seizure-like activity. Up to approximately 10 s, the number of future ephaptic mode cells (labeled as “6”) remains relatively low. By contrast, beyond 15,000 TUs (15 Sec.), the number of these cells increases dramatically to around 330, while the number of synaptically active cells is much lower. During this phase, the oscillation amplitudes in the system stabilize between 50 and 300, indicating that ephaptic conduction has become the dominant mode of neuronal activity. This shift suggests a clear transition from synaptic dominance to ephaptic dominance, which we interpret as corresponding to the progression from the tonic to the clonic phase of a seizure.

In this model, the entire run is assumed to represent the tonic–clonic transition, with synaptic conduction prevailing in the earlier tonic phase and ephaptic conduction taking over in the clonic phase.

What is particularly significant about this simulation is that no external interventions or additional material changes are required for the transition from tonic to clonic phases. This transition arises naturally from the interaction between synaptic and ephaptic conduction mechanisms within the model. The dynamic interplay between these two modes drives the system toward a tonic–clonic seizure state, with the synaptic mode primarily responsible for the chaotic activity of the tonic phase, and the ephaptic mode leading to the more organized, low-frequency oscillations characteristic of the clonic phase.

**Figure 4:**
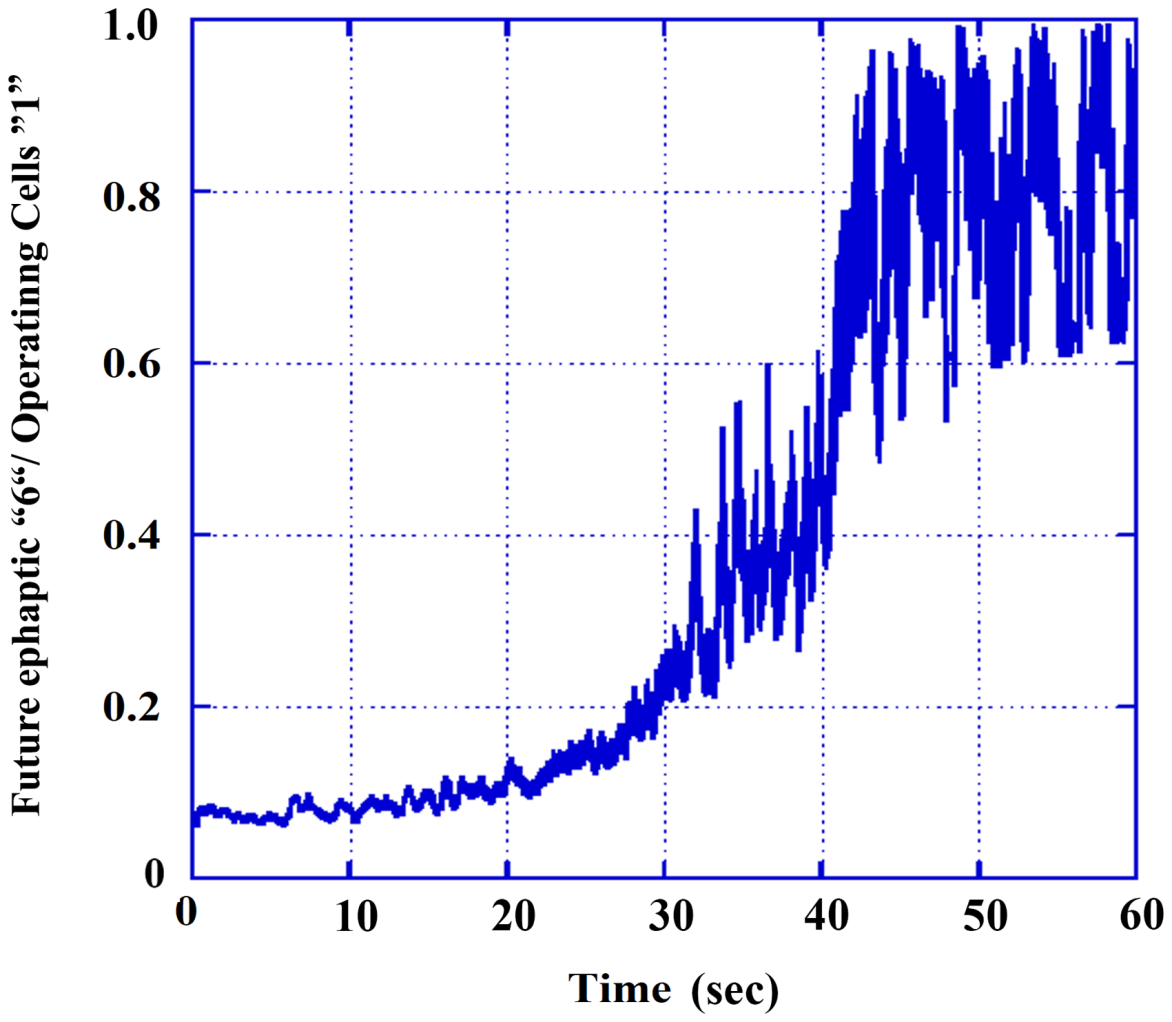
Ratio of the ephaptic mode out of the total activity. Smoothed values (moving averaged by 0.1 s) of part 3B (6′ s) divided by those of the total operating numbers (part 3A, 1′s).

To better understand the contribution of ephaptic conduction relative to overall neural activity, we calculated the ratio of cells operating in the ephaptic mode (labeled as “6’s” in Figure 3B) to the total number of active cells (labeled as “1’s” in Figure 3A). This ratio was smoothed using a moving average over 100 TUs to reduce fluctuations and highlight the general trend of the gradual evolution of ephaptic dominance over synaptic activity during the transition from the tonic to the clonic phase. The results are presented in Figure 4. It can be observed that the ratio of states increases from approximately 5% in the tonic phase to around ~90% in the clonic phase, and the transition itself is completed at ~40,000 TUs.

**Figure 5:**
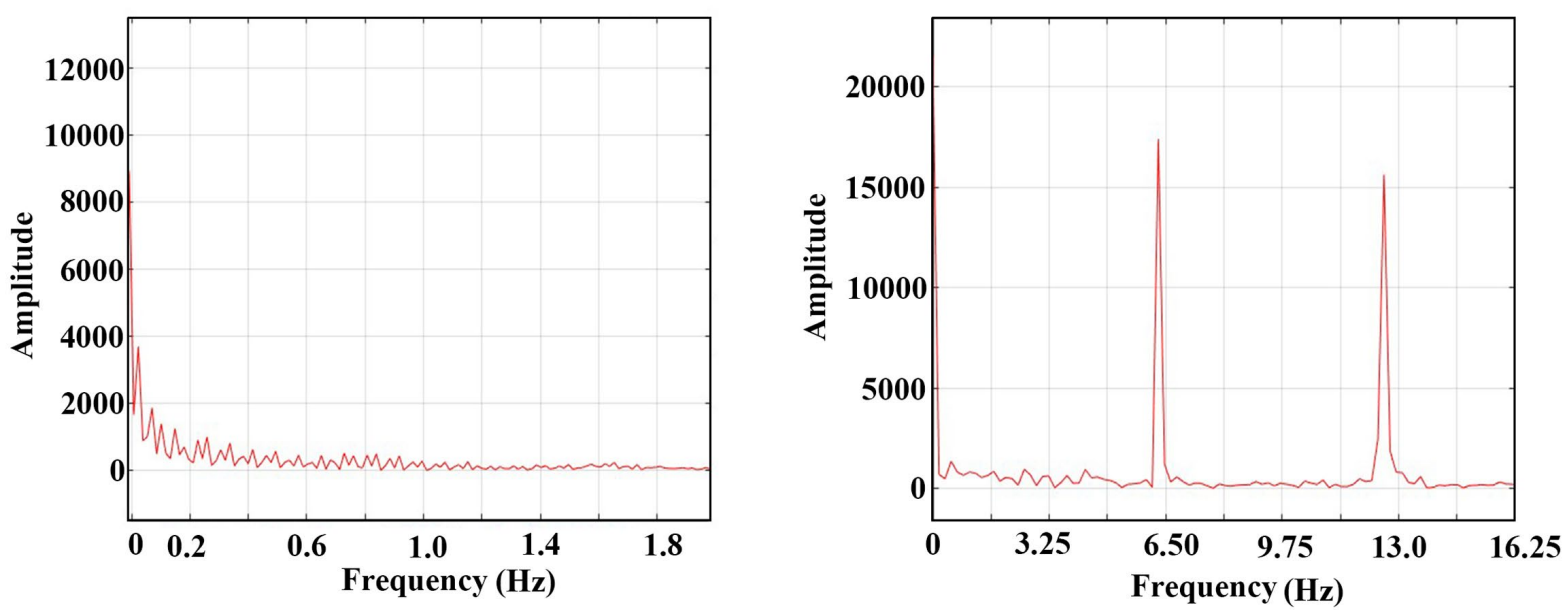
FFT of the domination transfer of the system from synaptic conduction to an ephaptic conduction one. Left: FFT of 0–1 s of Figure 3 A (synaptic domination region). Zero amplitude after 2 Hz. Right: FFT of 49–50 s (ephaptic domination region). One harmonic is shown.

The FFT (Fast Fourier Transform) analysis of the tonic and the clonic phases from Figure 3A are presented in Figure 5. The X-axis of the graph represents the frequency components of the oscillatory activity during the tonic (left graph) and clonic (right graph) phases. The results indicate that during the tonic phase, there are no dominant frequencies while the oscillations in the clonic phase exhibit a prominent frequency of ~6 Hz (its first harmonics is also presented) reflecting the internal dynamics of the system during the clonic phase,

The comprehensive overview indicates a transition from the synaptic operating stage, characterized by a predominantly chaotic spectrum to a more organized periodic regime dominated by ephaptic conduction. The ephaptic domain exhibits a stable and periodic pattern of activity.

Furthermore, the evolution of the various operational and waiting states across the entire population of cells within the matrix (delay 12 TUs, distinct from the configuration depicted in Figure 3) is illustrated in the accompanying video [The file mapV1 is provided as Supplementary material and also through the following link: https://physics.bgu.ac.il/~yaacov/File/mapV1.mp4, Colors legend to video mapV1: waiting cells: Blue (0); Dark red: operating cells (1); Red: first refractory (2); Other colors: rest of refractory]. This video provides a dynamic visualization of how the number of active and inactive cells fluctuates over time, reflecting the intricate interplay between synaptic and ephaptic mechanisms.

The video effectively illustrates the transition from a seemingly random distribution of bursts of activity occurring at various locations within the neural matrix to a more organized pulsation pattern observable around time unit (TU) 60. This transition is characterized by the emergence of distinct sources of regular pulsations, with at least two identifiable origins. Such a phenomenon suggests that the “regular” pulsations observed during the clonic phase may be generated locally, potentially preceding their propagation to other regions of the brain via neural conduction pathways.

Additionally, a second video (The file map2 is provided as Supplementary material and also through the following link: https://physics.bgu.ac.il/~yaacov/File/mapV2.mp4), which exclusively features operating cells, presents an alternative scenario where, at approximately TU 200, a localized patch of cells emerges as a single source of rhythmic pulses. This patch demonstrates the capacity to generate oscillatory activity that subsequently radiates throughout the entire matrix, highlighting the dynamic nature of neuronal interactions. These insights may further contribute to our understanding of the transition dynamics in neural circuits during regular and pathological conditions.

## Discussion

The primary objective of this study was to elucidate the transitions between the tonic and clonic phases during an active “grand mal” seizure, while intentionally excluding the processes related to seizure initiation and termination.

Two model-specific considerations must be addressed:

1. It is important to note that in our model, the “electrodes” capture multi-unit activity [MUA, see, e.g., (18)] rather than electroencephalography (EEG), as they specifically measure the activity of neurons in their immediate vicinity. In contrast, EEG reflects the summation of post-synaptic potentials across a broader area.
2. The range of ephaptic dominance illustrated in Figure 5 demonstrates a periodicity of ~6 Hz, which is around the clinical average frequency observed during the clonic phase of a seizure.

The initial inquiry regarding the emergence of an ephaptic conduction mode during an epileptic seizure is, “How could such a mode arise?” Typically, the electric fields generated by neuronal currents during normal brain operations are too weak to induce ephaptic conductivity. If this were not the case, it is conceivable that our entire behavioral repertoire would be governed by such a mode, leading to fundamentally altered neurophysiological dynamics. In this context, we hypothesize that during the tonic phase of a grand mal seizure, the excessive firing of numerous neurons generates an electric field sufficiently strong to facilitate the emergence of an ephaptic conduction mode. The feasibility of this hypothesis has been substantiated within the framework of our model.

Our findings suggest that the transition from the tonic to the clonic phase may be precipitated by an escalation of neuronal activity throughout the tonic stage of the seizure. According to our model, this increase in activity can induce a shift in the action potential (AP) conduction mechanism from a predominantly synaptic modality to one characterized by ephaptic interaction. This transition alters the operational dynamics of neuronal patterns, transforming them from a somewhat chaotic and potentially synchronized state into a more organized structure of AP oscillations, which can be effectively monitored through recordings from corresponding electrodes that capture repetitive spike activity.

The subsequent question pertains to the maintenance of the ephaptic mode during the clonic phase of the seizure, particularly considering that the overall neural activity does not appear to be as intense as during the tonic phase. Our results indicate that, under the influence of the ephaptic conduction mode, the neuronal activity does become markedly intensified, producing electric fields potent enough to stimulate neighboring neurons into action ephaptically. This mechanism effectively perpetuates the ephaptic mode, allowing it to sustain itself. Such self-propagation underscores the critical role of ephaptic interactions in the dynamic landscape of seizure activity.

In a homogeneous tissue environment, focal waves typically manifest as “target” waves propagating uniformly in all directions. However, the brain is characterized by inherent inhomogeneities that significantly influence wave dynamics, which can be categorized into at least two distinct types.

First, the neuronal population comprises various types, with the primary categories being excitatory and inhibitory neurons, particularly prominent in the central nervous system (CNS). The activity of inhibitory neurons limits the propagation of waves, creating a significant barrier to ectopic wave transmission. This widely accepted concept posits that, under unaided synaptic conduction, the spread of ectopic target waves within the brain parenchyma would be substantially impeded by the presence of inhibitory neurons. Conversely, it is proposed that these waves could propagate more effectively under a mixed synaptic-ephaptic conduction mode or purely ephaptic conduction. Our model examined this hypothesis (see section 1.iii of the Results), corroborating the assertion that ectopic wave propagation is indeed hindered by inhibitory structures during normal synaptic functioning. Consequently, it is challenging for ectopic waves be they target waves, spiral waves, or others-to propagate effectively within the parenchyma under standard conditions. In contrast, under conditions dominated by ephaptic conduction, where excitatory and inhibitory neurons are treated more equally, local periodic oscillations predominate and can be transported by waves aided by synaptic conduction.

Second, many brain regions exhibit a non-homogeneous architecture, possessing a preferential direction for action potential (AP) propagation. This structural anisotropy elucidates the findings of numerous studies indicating that wave propagation primarily occurs along two dominant directions, 180° apart, particularly within the cerebral cortex and the hippocampus [(14) and references therein]. This directional bias is pivotal in understanding the mechanisms of wave dynamics within the brain, as it underscores the complexities of neuronal interactions and the influence of structural properties on electrical signal transmission. Thus, the interplay between tissue homogeneity, neuronal types, and structural anisotropy plays a crucial role in shaping the propagation characteristics of focal waves within the brain, warranting further investigation to fully elucidate these intricate relationships.

In a recent publication (19), the authors propose that all phases of the epileptic process are mediated by target (circular) waves originating from a specific focal point. This origin can be identified through phase differences in electrical potentials recorded by multiple electrodes placed at various locations within the brain. The study suggests that these waves propagate at speeds of approximately 0.3 m/s. Notably, the results concerning the localization of these wave sources exhibit a strong correlation with the success of brain resection surgeries aimed at reducing seizure frequency in several patients with epilepsy.

However, there is a notable discrepancy in the reported propagation speeds of these waves. Reference (19) indicates a wave speed of 0.3 m/s, whereas other studies, such as those referenced in (10, 11), report speeds of around 0.1 m/s. We posit that this difference may be attributed to variations in the timing of measurements across these studies. Specifically, we hypothesize that during the initial phases of a seizure (the evolutionary or tonic phase)-when measurements in (19) were presumably obtained, waves, which may be less easily observable at that stage, could travel at a faster speed of 0.3 m/s compared to waves observed during the later clonic phase, as recorded in references (10, 11), where the speeds appear to decrease to 0.1 m/s due to the influence of ephaptic conduction mechanisms.

Furthermore, the interpretation presented in reference (19), which describes the gradual phase shift between two electrodes as indicative of a change in the wave origin, could also be explained by the observed differences in wave propagation speeds. It is important to note that, while the initiation of a seizure may likely be governed by a single wave, as indicated by the phase delays observed in the EEG bursts among the electrodes (19), distinguishing additional waves during the tonic stages could prove challenging. However, distinct wave successions are more readily observable during the clonic stages of the seizure, providing further insights into the complex dynamics of epileptic activity.

We argue that the simultaneous operation of both synaptic and ephaptic conductivity modes within the brain is feasible when a sufficient number of neurons are concurrently active, thereby generating an electric field strong enough to enable the ephaptic effect alongside the synaptic interactions. Such a scenario is particularly plausible during an epileptic seizure, where a large population of neurons fires synchronously, allowing the ephaptic effect to dominate, as demonstrated both experimentally [e.g., (15)] and theoretically within our model.

The mechanism underlying this ephaptic dominance is readily comprehensible. It stems from the rapid transmission speed—approximating the speed of light—at which the information conveyed by the electromagnetic field reaches the neurons, significantly surpassing the “chemical” transmission speed associated with synaptic effects. In our model, this disparity in information transmission speed is represented by the prioritized response of neurons to ephaptic stimuli.

Furthermore, it is essential to acknowledge that, despite the rapidity of ephaptic signaling, effective generation of an action potential (AP) can only occur when the amplitude of the ephaptic influence surpasses a specific threshold. When this influence is sufficiently strong (i.e., above the threshold), it can dominate overall neuronal electrical conduction. In instances characterized by intermediate levels of ephaptic influence, this effect may manifest prominently in certain regions of the brain while remaining attenuated in others, leading to heterogeneous patterns of neuronal electrical conduction.

The propagation of slow waves during the clonic phase is facilitated by NMDA receptor channels, as discussed in reference (11), and is further elucidated through mathematical modeling in reference (16).

The presence of ephaptic conduction during tonic–clonic seizures carries significant implications for the treatment of epilepsy. Two primary considerations arise:

(1) Our model provides a more comprehensive explanation for the relative ineffectiveness of transection treatments for this disorder, as noted by reference (10). For instance, following surgical intervention, the success of the treatment is contingent upon its ability to halt the seizure during its initial (tonic) phase, which primarily develops through synaptic conduction. Surgical incisions can impede the spread of this phase; however, if a sufficiently large segment of the brain remains between the incisions, it may allow the tonic part of the seizure to develop locally. While this confinement may be advantageous for the patient by preventing the seizure from synaptically spreading to other brain areas, if the oscillatory activity within this segment intensifies enough to trigger the clonic phase, the treatment would become ineffective. This ineffectiveness arises because the incisions *cannot interrupt* the progression of the seizure during the clonic phase, which is propagated by the ephaptic mode that *can* transcend these surgical barriers, as highlighted in references (10, 11). Moreover, in cases of non-synaptic epileptogenesis, as discussed in reference (20), such treatments may not yield successful outcomes.
(2) Recent advancements in non-invasive brain stimulation systems, utilizing magnetic coils in both open-loop (20) and closed-loop (21) configurations, have been proposed as promising therapeutic approaches. These systems have the potential to disrupt ephaptic transmission, thereby offering the possibility of reducing the duration of tonic–clonic seizures and potentially halting seizures that are initiated by ephaptic epileptogenesis. Such seizures can arise under excess excitability conditions, if there appears to be a surplus of neuronal functioning, even locally.

## Data Availability

The raw data supporting the conclusions of this article will be made available by the authors, without undue reservation.
